# Comparison of Molecular Potential for Iron Transfer across the Placenta in Domestic Pigs with Varied Litter Sizes and Wild Boars

**DOI:** 10.3390/ijms25179638

**Published:** 2024-09-05

**Authors:** Zuzanna Kopeć, Rafał Radosław Starzyński, Małgorzata Lenartowicz, Małgorzata Grzesiak, Jolanta Opiela, Zdzisław Smorąg, Barbara Gajda, Jakub Nicpoń, Magdalena Ogłuszka, Xiuying Wang, Rafał Mazgaj, Adrian Stankiewicz, Wiktoria Płonka, Natalia Pirga-Niemiec, Sylwia Herman, Paweł Lipiński

**Affiliations:** 1 Department of Molecular Biology, Institute of Genetics and Animal Biotechnology, Polish Academy of Sciences, 05-552 Jastrzębiec, Poland; z.kopec@igbzpan.pl (Z.K.);; 2 Laboratory of Genetics and Evolution, Institute of Zoology and Biomedical Research, Jagiellonian University, 31-007 Kraków, Poland; 3 Department of Endocrinology, Institute of Zoology and Biomedical Research, Jagiellonian University, 31-007 Krakow, Poland; 4 National Research Institute of Animal Production, 32-083 Balice, Poland; 5 Department of Surgery, Faculty of Veterinary Sciences, Wrocław University of Environmental and Life Sciences, 50-375 Wrocław, Poland; 6 Department of Genomics, Institute of Genetics and Animal Biotechnology, Polish Academy of Sciences, 05-552 Jastrzębiec, Poland

**Keywords:** placenta, iron deficiency anemia (IDA), litter size, copper-dependent ferroxidases, domestic pig, wild boar

## Abstract

Neonatal iron deficiency anemia is prevalent among domestic pigs but does not occur in the offspring of wild boar. The main causes of this disorder in piglets of modern pig breeds are paucity of hepatic iron stores, high birth weight, and rapid growth. Replenishment of fetal iron stores is a direct result of iron transfer efficiency across the placenta. In this study, we attempted to investigate the molecular potential of iron transfer across the placenta as a possible cause of differences between wild boar and Polish Large White (PLW) offspring. Furthermore, by analyzing placentas from PLW gilts that had litters of different sizes, we aimed to elucidate the impact of the number of fetuses on placental ability to transport iron. Using RNA sequencing, we examined the expression of iron-related genes in the placentas from wild boar and PLW gilts. We did not reveal significant differences in the expression of major iron transporters among all analyzed placentas. However, in wild boar placentas, we found higher expression of copper-dependent ferroxidases such as ceruloplasmin, zyklopen, and hephaestin, which facilitate iron export to the fetal circulation. We also determined a close co-localization of ceruloplasmin and zyklopen with ferroportin, the only iron exporter.

## 1. Introduction

Early postnatal iron-deficiency anemia (IDA) is a common disorder in mammals, but it is consistently observed only in domestic pigs [1,2]. Numerous factors contribute to IDA in piglets of contemporary high-performance breeds, with key determinants including low hepatic iron stores, high birth weight, and rapid piglet growth [1,3]. Considering that iron reserves constitute a major source of this microelement for the development of mammalian neonates [4], it seems that the low content of hepatic iron (presumably the lowest among mammalian newborns [1,5]) accumulated during the fetal period is an initial and primary cause of IDA in newly born piglets. It has long been thought that this phenomenon is due to the physiological inability of pregnant sows to deliver sufficient amount of iron to fetal livers, and thus to meet the iron demand for the growing number of fetuses. Indeed, reproductive efficiency is an important economic goal in the swine industry, and the selection for increased litter size over the past few decades has been one of the main objectives in pig breeding in many countries [6,7,8]. Interestingly, the results of our recent study clearly demonstrate that varying the number of Polish Large White (one of the two principal maternal pig breeds in Poland) piglets in a litter by embryo transfer does not affect the hepatic iron content and red blood cell (RBC) status of one-day-old piglets, all showing signs of iron deficiency [9]. We also presented the first experimental evidence that RBC status in wild boar piglets is higher than that in PLW piglets, regardless of the litter size they come from [9]. The optimal transfer of iron across the placenta is essential for fetal development in utero and for the establishment of adequate birth iron stores to sustain growth in early infancy. In this context, a question arises about the potential limitations in the functioning of complex mechanisms of iron transport across the placenta in domestic pig females. Therefore, in the present study, we attempted to compare the expression pattern of iron-related genes in the placenta between wild boar and PLW females of different litter sizes, including small litters equivalent to typical wild boar ones. Multiple maternal, placental, and fetal regulatory mechanisms are involved in the control of iron transport through the placenta [10,11]. Animal model studies indicate that especially in conditions of iron deficiency during pregnancy, iron is preferentially delivered, at the expense of the mother, to maintain not only fetal growth but also iron-dependent placental processes that are indispensable for the overall functioning of this organ [12,13]. In the term mouse/human placenta, the unidirectional iron flux to the fetal circulation is mediated by polarized syncytiotrophoblasts (SCTB) [14,15]. Diferric transferrin (Tf-Fe_2_) from the maternal circulation binds to transferrin receptor 1 (TfR1) located on the apical (maternal) surface of syncytiotrophoblasts (SCTB) and is taken up by these cells via clathrin-mediated endocytosis. Inside the cell, specialized endosomes are formed and subsequently acidified by a proton pump. At pH 5.5, ferric (Fe^3+^) iron is released from transferrin molecules, reduced to the ferrous state (Fe^2+^) by an oxidoreductase (potentially Steap3) [16], and is then transported to the cytoplasm via divalent metal transporter 1 (DMT1) [17]. Once in the cytoplasm, iron can be stored in ferritin (Ft) [18], used for iron–sulfur cluster biogenesis and heme synthesis, or exported to the fetal circulation by ferroportin (Fpn) [19]. Iron export from SCTB requires one of the multicopper ferroxidases, with zyklopen (Zp), hephaestin (Heph), and/or ceruloplasmin (Cp) being likely candidates, which oxidize Fe^2+^ to Fe^3+^ prior to iron binding by fetal transferrin in the blood [20,21].

The aim of our study was to compare the molecular mechanisms of placental iron transfer in wild boar and Polish Large White (PLW) females, particularly in the context of neonatal iron deficiency anemia (IDA) occurring in domestic pigs but not in wild boars [9]. Additionally, we aimed to check whether a large number of piglets in the litter of domestic pig females entails enhancement of transplacental iron transport. We sought to solve this issue by analyzing the expression of iron-related genes in the placentas of PLW females with different-size litters.

Although neonatal IDA in domestic piglets is well documented [1,2,3], there are no studies focused on the efficiency of molecular mechanisms of transplacental iron transfer as a potential causative factor underlying this phenomenon. Our knowledge of molecular mechanisms involved in placental iron transfer in *Sus* species is very poor [22]. In particular, their impact on the occurrence of IDA in suckling piglets of modern breeds remains unclear. We hypothesize that the potential of transplacental iron transfer in wild boar females shaped during evolution remained unchanged in domestic pig females of contemporary high-performance breeds and turned out to be insufficient to provide enough iron to a larger number of heavier piglets.

In this study, we employed RNA sequencing (RNA-seq) to compare for the first time the expression of genes involved in transplacental iron transfer between wild boar and PLW females with varying litter sizes. Our study provides new insights into the role of copper-dependent ferroxidases, such as ceruloplasmin, zyklopen, and hephaestin, in iron export efficiency to fetal circulation. RNA-seq data, confirmed by RT quantitative PCR (RT-qPCR), indicated that among iron-related genes, only those encoding multicopper ferroxidases were highly expressed in wild boar placentas compared to those in PLW placentas. Immunofluorescence analysis revealed enhanced fluorescent signals for ceruloplasmin (Cp) in the apical membrane of the luminal epithelial cells in the wild boar placenta. Co-localization studies showed overlap in the staining of Cp and zyklopen (Zp) with ferroportin (Fpn), the known exporter of non-heme iron [11,23].

Although we did not detect any major differences in the molecular potential to transfer iron through the placenta between wild boar and domestic PLW pig, our data suggest that especially Cp may contribute to more efficient iron flow across the placenta in wild boars compared to that in PLW females, regardless of their litter size.

## 2. Results

### 2.1. NGS-Based RNA-Seq Analysis of Wild Boar and PLW Placentas from Females Having Litters of Different Sizes

Through the analysis of RNA-seq data, we identified statistically significant differences in gene expression between the placentas of the wild boar and domestic pig groups. Volcano plots were employed to delineate genes with expression changes exceeding a fivefold difference between the placentas of wild boars and domestic pigs groups (Figure S1A–F). Among placentas of wild boars and PLW females with small litters after embryo transfer (ET), we observed differential expression in 164 genes, with 138 upregulated and 26 downregulated. Pronounced expression disparities were noted between the placentas of wild boar and PLW females with large litters after ET, involving 402 genes, with 94 upregulated and 308 downregulated. Conversely, the least variation in gene expression occurred between the placentas of wild boars and PLW females with large litters after artificial insemination (AI), encompassing 82 genes, with 46 upregulated and 36 downregulated. Regarding gene expression differences in domestic pigs with different litter sizes, only singular alterations were noted: one gene was downregulated in the large litter (AI) vs. small litter (ET), one gene was upregulated in the large litter (AI) vs. large litter (ET), and one gene was downregulated (Table 1).

The RNA-seq data analysis revealed significant differences in gene expression between the placentas of wild boar and PLW females, with the most pronounced changes observed between wild boar and PLW females having large litters obtained through embryo transfer (ET). In contrast, differences in gene expression among PLW females with different litter sizes were minimal, with only a few single-gene expression changes noted between domestic pig groups.

### 2.2. Lack of Major Changes in the Placental Expression of Main Iron Transporters and L-Ferritin between Wild Boar and PLW Gilts

**Figure 1:**
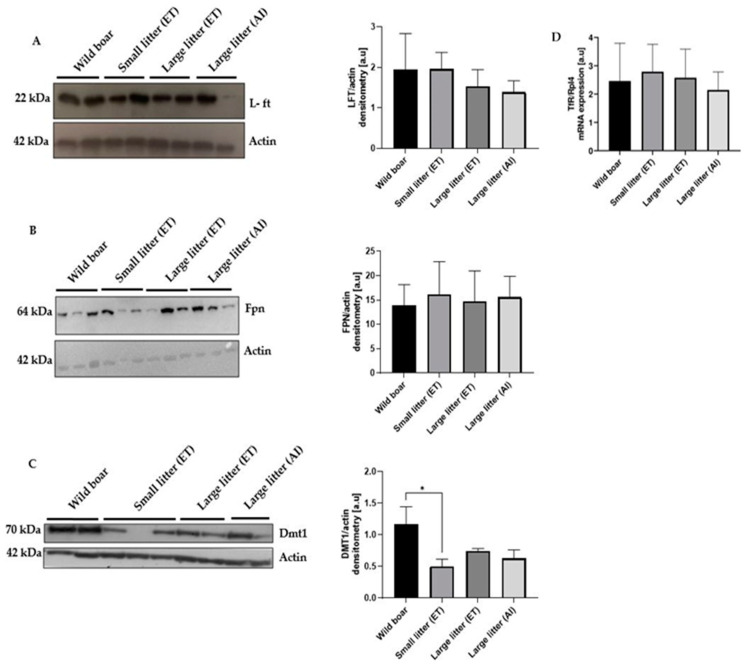
Expression of key iron transporters and L-ferritin in the placentas of wild boar and Polish Large White females with different-size litters. (**A**) Light chain ferritin (L-Ft), (**B**) ferroportin (Fpn), and (**C**) divalent metal transporter 1 (DMT1) in placenta protein extracts were assessed by Western blot analysis. The blots were also re-probed with monoclonal anti-β-actin antibody from mouse as a loading control. Right-hand panels, the intensity of L-Ft, Fpn, and DMT1 bands was quantified with a molecular Imager using Quantity One 4.6 software (Bio-Rad, Hercules, California USA) and is plotted in arbitrary units (a.u.) to present protein levels (means ± S.D.). (**D**) Transferrin receptor 1 (TfR1) mRNA level in the placenta measured by real-time quantitative PCR using specific primer pairs (as shown in Table S2). The ribosomal protein L4 (Rpl4) amplicon was used as a control for the amount of cDNA used in the PCR reactions. Each column represents the means ± S.D. of two amplification reactions, performed using a single cDNA sample reverse-transcribed from RNA prepared from 4–6 females. Significant differences are indicated (* *p* < 0.05).

Although our RNA-seq analysis did not reveal any statistically significant differences in the expression of genes encoding for iron transporters and ferritin between the placentas from different experimental groups (the log2FoldChange values showed minimal variation, and the corresponding adjusted *p*-values (padj) obtained from the DESeq analysis were above 0.05), we considered it important to assess their protein and/or mRNA levels by Western blotting and/or RT-qPCR. The protein levels of L-ferritin (L-Ft) (Figure 1A) and ferroportin (Fpn) (Figure 1B) in the placenta showed no significant differences between wild boar and PLW mothers. In contrast, wild boar females exhibited higher placental DMT1 protein levels, but only in comparison to PLW gilts having small litters after ET (Figure 1C). The expression of transferrin receptor 1 (TfR1), responsible for iron loading to the placenta from the maternal circulation, assessed at the mRNA level by RT-qPCR, showed no statistically significant differences between all examined groups (Figure 1D).

Although the RNA-seq analysis did not reveal significant differences in the placental expression of most iron transporters and ferritin, protein levels of DMT1 were higher in wild boars compared to that in PLW females with small litters, while no significant differences were found in L-ferritin, ferroportin, or transferrin receptor 1 expression.

### 2.3. Increased Expression of Multicopper Ferroxidases in Wild Boars Compared to That in PLW Placenta

**Figure 2:**
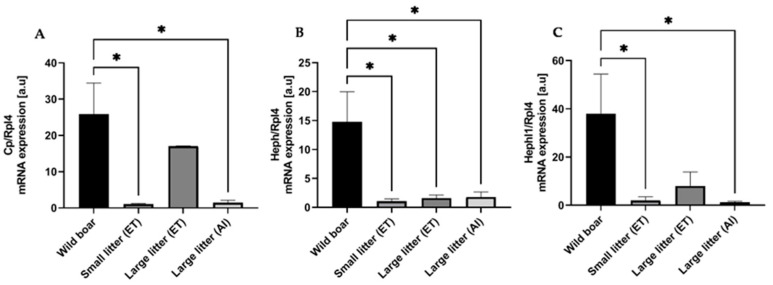
Increased expression of (**A**) ceruloplasmin (Cp), (**B**) hephaestion (Heph), and (**C**) hephaestin like 1 (Hephl1) mRNA in the placenta of wild boars compared to that in Polish Large White females with different-size litters. Transcript abundance in placentas was measured by real-time quantitative PCR using specific primer pairs (as shown in Table S2). The ribosomal protein L4 (Rpl4) amplicon was used as a control for the amount of cDNA used in the PCR reactions. Each column represents the means ± S.D. of two amplification reactions, performed using a single cDNA sample reverse-transcribed from RNA prepared from 4–6 females. Significant differences are indicated (* *p* < 0.05).

Interestingly, our RNA-seq data implied that genes encoding for multicopper ferroxidases, such as ceruloplasmin (Cp), hephaestin (Heph), and zyklopen (Zp), that are essential for iron efflux from tissues [20,21] are highly upregulated in wild boar placentas compared to those in placentas from PLW females, regardless of their litter size. The respective log2FoldChange and corresponding adjusted *p*-value (padj) for all comparison axes of three examined genes are shown in Table 2. In parallel, we did not detect any differences in the placental expression of *Hephl1* (encoding for Zp), *Cp*, and *Heph* genes among PLW gilts having litters of different sizes. To validate the results of RNA-seq analysis, we analyzed *Zp*, *Cp*, and *Heph* mRNA levels by RT-qPCR. Our results largely confirmed the RNA-seq data and revealed a markedly increased expression of all three genes in the wild boar placentas compared to those of PLW gilts with different-size litters (Figure 2A–C). The only not statistically significant difference was found in the *Cp* mRNA levels between wild boar and PLW gilts having large litters after ET (Figure 2A). Interestingly, no differences were detected in the expression of ferroxidase genes between PLW gilts from all experimental groups. These results clearly show that high expression of multicopper ferroxidases at the mRNA level in the wild boar placenta is not preserved in PLW females, and in the latter, it does not depend on the number of piglets in the litter.

Our RNA-seq and RT-qPCR analyses demonstrated that multicopper ferroxidases, such as ceruloplasmin, hephaestin, and zyklopen, were significantly upregulated in the placentas of wild boars compared to those in PLW females, regardless of litter size. In contrast, no differences were observed in the expression of these ferroxidase genes among PLW females with varying litter sizes, indicating that high expression levels in wild boar placentas are not retained in domestic pigs and do not depend on litter size in the latter.

### 2.4. Placental Copper Content and Expression of Copper-Related Genes in Wild Boar Sows and PLW Gilts with Different-Size Litters

**Figure 3:**
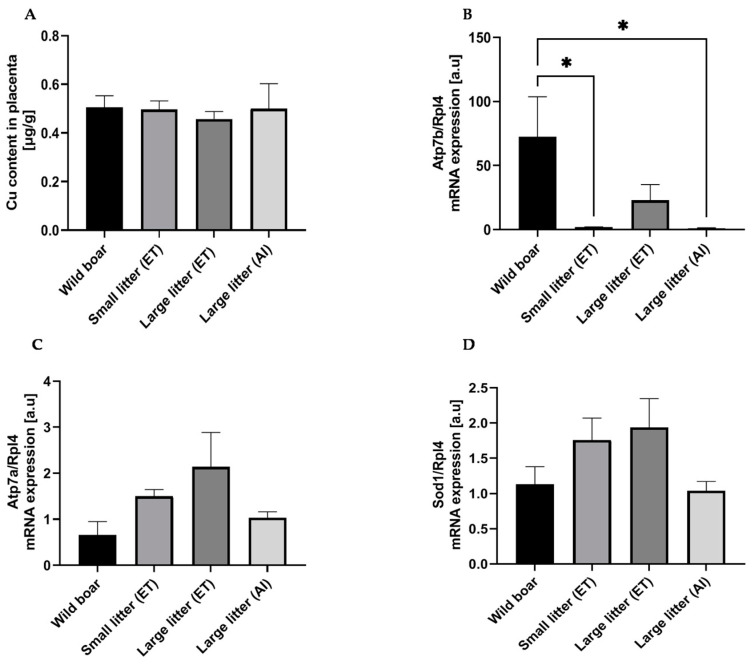
Placental copper content and expression of copper-related genes in wild boar sows and Polish Large White gilts with different-size litters. (**A**) Total copper content in the placenta measured by atomic absorption spectroscopy as described in the Materials and Methods. The values are expressed as the mean ± S.D. and were obtained from 5 or 6 females. (**B**) Atp7b, (**C**) Atp7a, and (**D**) superoxide dismutase 1 (Sod1) mRNA levels in the placenta. Transcript abundance in the placentas was measured by real-time quantitative PCR using specific primer pairs (as shown in Table S2). The ribosomal protein L4 (Rpl4) amplicon was used as a control for the amount of cDNA used in the PCR reactions. Each column represents the means ± S.D. of two amplification reactions, performed using a single cDNA sample reverse-transcribed from RNA prepared from 4–6 females. Significant differences are indicated (* *p* < 0.05).

Fluctuations in copper content may influence the expression and function of multicopper ferroxidases [24]. Having demonstrated their higher expressions in the wild boar placenta, we decided to measure and compare the placental copper level between all experimental groups, assuming that elevated copper levels in the wild boar placenta may be a causative factor of the increased expression of examined ferroxidases. However, we found that the copper content in placentas from wild boar and PLW gilts regardless of litter size was similar, without any significant differences (Figure 3A). Then, we checked whether the phenomenon of increased multicopper ferroxidases expression in the wild boar placenta extended to other enzymes containing copper in their functional redox centers. We analyzed the expression of superoxide dismutase 1 (*Sod1*), an antioxidant enzyme [25], and two P-type ATPases, *Atp7a* and *Atp7b*, which play crucial but distinct roles in copper metabolism [26]. We did not find any differences neither in *Sod1* nor in *Atp7a* mRNA abundance between the experimental groups (Figure 3D and Figure 3C, respectively). Interestingly, the expression of the *Atp7b* gene was several-fold higher in the placenta of wild boars compared to that in the placenta of PLW females having small litters after ET, large litters after AI, and only approximately three times higher (not statistically significant difference) compared to that in gilts having large litters after ET (Figure 3B). Considering that Atp7b-mediated incorporation of copper into apo-Cp results in the formation of the stable redox-active holoenzyme [27], an orchestrated increase in the expression of *Cp* and *Atp7b* genes points to Cp as the main ferroxidase acting in the wild boar placenta.

Despite higher expressions of multicopper ferroxidases in wild boar placentas, copper content and the expression of other copper-containing enzymes, such as Sod1 and Atp7a, did not differ significantly between wild boar sows and domestic PLW gilts. Notably, Atp7b expression was significantly higher in wild boar placentas. 

### 2.5. Immunofluorescent Localization of Multicopper Ferroxidases and Their Co-Localization with Ferroportin (Fpn) in the Maternal Part of the Placenta of Wild Boar and PLW Females

**Figure 4:**
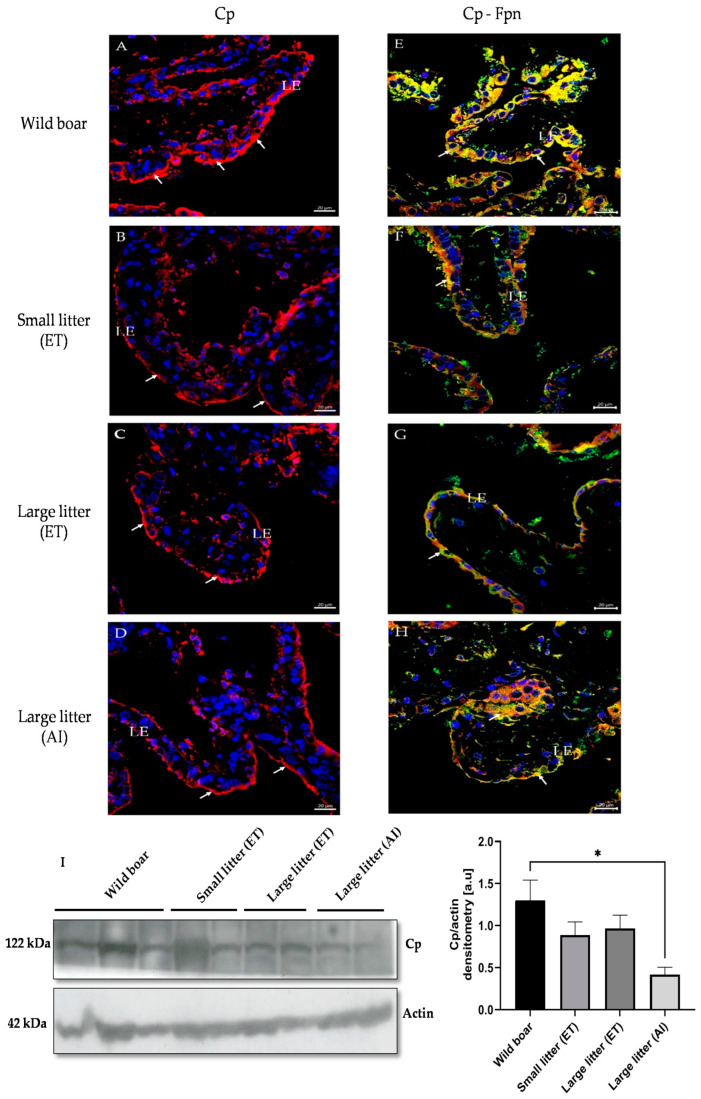
Immunofluorescent localization of ceruloplasmin (Cp) in the maternal part of the placenta of (**A**) wild boar females and Polish Large White gilts having: (**B**) small litters obtained by embryo transfer (ET), (**C**) large litters obtained by ET, and (**D**) large litters obtained by artificial insemination (AI). (**E**–**H**) Respective co-localization of Cp with ferroportin (Fpn). In the left-hand panels, the expression of Cp detected by confocal microscopy was found as a positive immunofluorescent signal (red channel) in the apical membrane of the luminal epithelial (LE) cells of the endometrium (white arrows). In the right-hand panels, the co-localization of Cp (red channel) and Fpn (green channel) is shown in the merged image. Cp–Fpn complexes were visible in the apical membrane of LE (white arrows). The location of the nuclei is disclosed by counterstaining with DAPI (blue channel). Scale bars correspond to 20 μm. (**I**) Cp protein levels in placenta membrane extracts were assessed by Western blot analysis. The blots were also incubated with a monoclonal anti-β-actin antibody from mouse as a loading control. In the right-hand panel, the intensity of Cp bands was quantified with a molecular Imager using Quantity One 4.6 software (Bio-Rad) and is plotted in arbitrary units (a.u.) to present protein levels (means ± S.D.). Significant differences are indicated (* *p* < 0.05).

**Figure 5:**
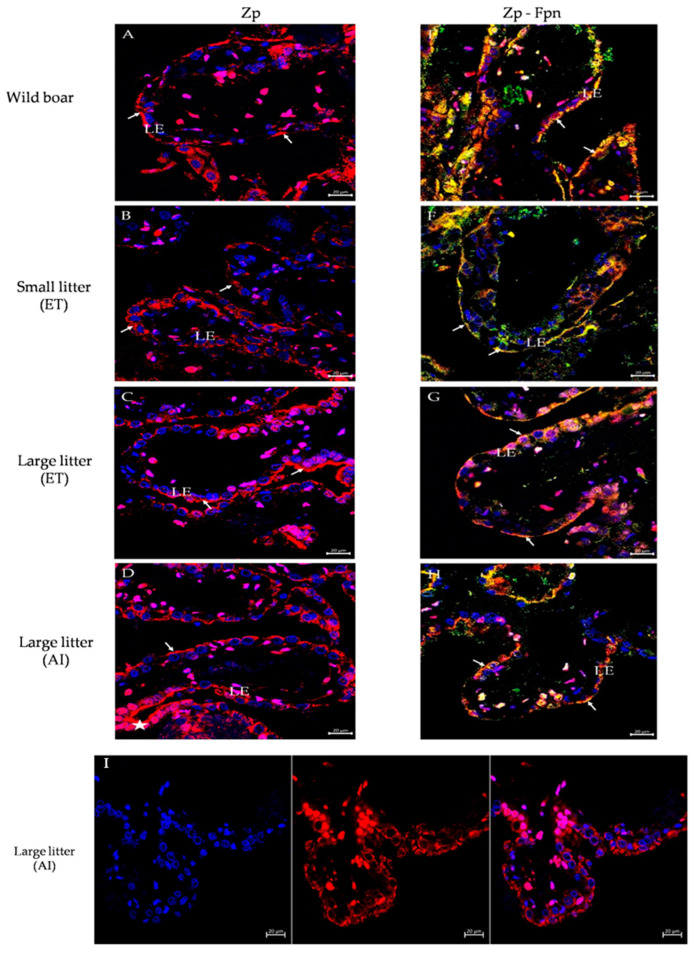
Immunofluorescent localization of zyklopen (Zp) in the maternal part of the placenta of (**A**) wild boar females and Polish Large White gilts having: (**B**) small litters obtained by embryo transfer (ET), (**C**) large litters obtained by ET, and (**D**) large litters obtained by artificial insemination (AI). (**E**–**H**) Respective co-localization of Zp with ferroportin (Fpn). In the left-hand panels, the expression of Zp detected by confocal microscopy was found as a positive immunofluorescent signal (red channel) in the apical membrane of the luminal epithelial (LE) cells of the endometrium (white arrows). In the right-hand panels, the co-localization of Zp (red channel) and Fpn (green channel) is shown in the merged image. Zp–Fpn complexes were visible in the apical membrane of LE (white arrows). Additionally, we found nuclear Zp protein localization (white star) within LE cells (**D**). This observation is detailed in (**I**): in the left-hand panel, the location of the nuclei is disclosed by counterstaining with DAPI (blue channel); in the middle panel, there is a Zp-positive immunofluorescent signal (red channel) in the luminal epithelial cells (LE) in the basal part of the placental villi; and in the right-hand panel, the location of Zp (red channel) in the nuclei (DAPI, blue channel) of these cells is shown in the merged image. The presence of Zp in the nuclei is visualized on placenta sections from PLW gilts having large litters after AI; however, it is representative of placentas from all experimental groups. Scale bars correspond to 20 μm.

**Figure 6:**
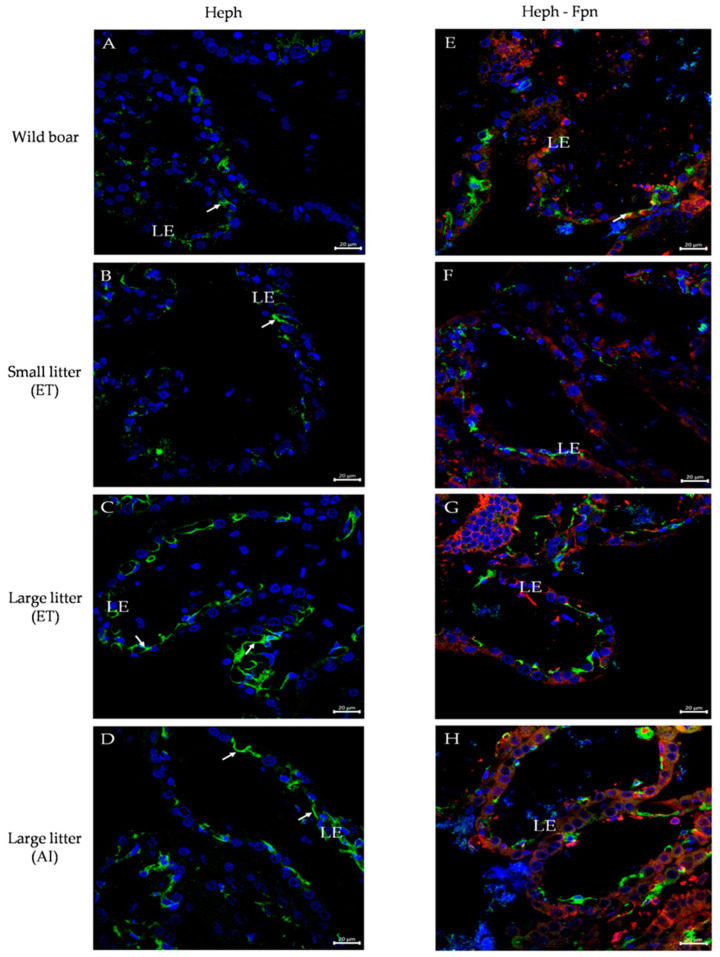
Immunofluorescent (IF) localization of hephaestin (Heph) in the maternal part of the placenta of (**A**) wild boar females and Polish Large White gilts having: (**B**) small litters obtained by embryo transfer (ET), (**C**) large litters obtained by ET, and (**D**) large litters obtained by artificial insemination (AI). (**E**–**H**) Respective co-localization of Heph with ferroportin (Fpn). In the left-hand panel, the expression of Heph detected by confocal microscopy was found as a positive immunofluorescent signal (green channel) in the basal membrane of the luminal epithelial (LE) cells of the endometrium (white arrows). In the right-hand panels, the co-localization of Heph (green channel) and Fpn (red channel) was only found in a few LE cells (white arrow) in the placentas of wild boars (**E**). Heph–Fpn co-localization was not identified in placenta sections from all PLW groups (**F**–**H**). Cell nuclei were counterstained with DAPI (blue channel). Scale bars correspond to 20 μm.

To assess the potential involvement of multicopper ferroxidases in transplacental iron transport, we examined their localization in the maternal part of the placentas from wild boar and PLW females. Considering that in mammalian cells the main pathway for cellular iron egress relies on the cooperation of multicopper ferroxidases with ferroportin (Fpn), the only known iron exporter [23], we performed double immunolocalizations of Cp, Zp, and Heph with Fpn. First, in wild boar and PLW females, we observed a marked staining of Cp in the maternal part of the placenta, specifically in the apical membrane of luminal epithelial cells of the endometrium (Figure 4A–D). A particularly strong Cp immunopositive signal was detected in the placenta of wild boar females (Figure 4A). Co-localization analysis revealed a tight overlap in the staining of Cp and Fpn (Figure 4E–H) suggesting that the Cp–Fpn system promoting iron release from cells is functional in both wild boar and PLW placentas. To provide further evidence of the presence of Cp in placentas from all experimental groups, we assessed Cp levels by Western blotting, showing a clear tendency to a higher level in the placenta of wild boars compared to that in PLW females (Figure 4I). Zp, considered the main placental ferroxidase [28], was found similarly to Cp, in the apical membrane of luminal epithelial cells of the maternal part of wild boar and PLW placentas (Figure 5A–D), and showed tight co-localization with Fpn (Figure 5E–H). An immunopositive Zp signal was similar in the placentas from all examined groups. Surprisingly, in both wild boar and PLW groups, we also detected the presence of Zp protein expression in the nuclei of the luminal epithelial cells exclusively in the basal part of the placental villi (Figure 5D, white star and Figure 5I). The nuclear localization of Zp was attested by double immunolocalization with DAPI, a specific marker for DNA (Figure 5I). Finally, using the immunofluorescence method, we analyzed the placental localization of Heph, a trans-membrane protein, which plays a critical role in iron absorption by duodenal enterocytes [29], although its expression has also been found in the mouse placenta [14]. Heph-positive staining (showing similar signal intensity in wild boars and in PLW placentas) was observed in some but not all luminal epithelial cells of the maternal part of the placenta. In contrast to Cp and Zp, the expression of Heph was mainly found in the basal membrane of the luminal epithelial cells (Figure 6A). Only in the wild boar placenta, a few cells revealed the co-localization of Heph and Fpn in the apical membrane (Figure 6E). This Heph–Fpn co-localization was not found in any PLW group (Figure 6F–H).

Our immunolocalization analyzes demonstrated that Cp and Zp were prominently expressed in the apical membrane of luminal epithelial cells in the maternal part of the placenta in both wild boar and PLW females, with Cp showing particularly strong expression and co-localizing with Fpn in wild boars. In contrast, while hephaestin (Heph) was detected in both wild boar and PLW placentas, its expression was limited to the basal membrane and showed co-localization with Fpn only in wild boars, suggesting a distinct role for Heph in iron transport between these species.

## 3. Discussion

The placenta serves as the interface between the mother and fetus, mediating nutrient transport to the latter, including the transport of iron. The unidirectional transfer of iron taken up by the placenta from the maternal to the fetal circulation is the only pathway to satisfy the iron requirements of the fetus and to build up fetal hepatic iron stores, which are thought to be the major source of iron during neonatal development [4]. Critically low fetal/newborn hepatic iron stores are considered a primary cause of neonatal iron deficiency anemia (IDA) in modern breeds of domestic pig (*Sus scrofa domestica*) [1,2,3]. However, it is largely unknown to what extent insufficient placental iron transfer contributes to this fetal iron paucity. In this context, it is worth noting that attempts to supplement pregnant sows with iron to enhance iron transfer across the placenta, fortify the iron stores in their fetuses, and consequently prevent IDA in newborns have been largely unsuccessful [30]. Moreover, our recent results demonstrated that varying the number of piglets in a litter of domestic pigs (from 4 to 14 animals) does not affect the iron status of one-day-old piglets, with all showing IDA [9]. These results suggested that iron delivery across the pig placenta is set at a relatively low level, which excludes enhanced iron transport and the improvement of iron status of piglets from small litters. Additionally, in the same study, we demonstrated that wild boar piglets (typically from litters of 4–6 animals) show similar iron content in the liver compared to that in domestic pig piglets (regardless of litter size) but do not show any symptoms of IDA [9]. These observations prompted us to compare the expression of genes involved in iron trafficking across the placenta between wild boar and domestic pig gilts with different litter sizes. For this purpose, we used placentas from wild boar and PLW gilts with small and large litters, obtained through regulated embryo transfer (ET) or artificial insemination (AI), as recently described [9].

The molecular basis of iron flow through the placenta in pigs is very poorly understood. Reports from the 1980s proposed that uteroferrin, an iron-binding glycoprotein possessing acid phosphatase activity, synthesized and secreted by the maternal uterus, is involved in iron transport to the fetus [31,32]. However, it was later shown that direct transfer of iron to apotransferrin is unlikely to be a physiological role of uteroferrin [33]. Recently, a role of placental hepcidin and ferroportin in local placental iron transfer regulation for improving the iron reserve of piglets has been suggested [22].

To identify iron-related genes that are differentially expressed in the examined placentas, we performed RNA sequencing (RNA-seq) of placenta samples collected from wild boar sows and PLW gilts. RNA-seq data revealed no differences in the transcriptional pattern of iron-related transcripts between placentas of PLW gilts having different-size litters, strongly suggesting that the number of piglets in the litter of PLW gilts has no impact on placental iron metabolism, including iron transfer through the placenta. Similarly, among all genes differentially expressed in wild boar and PLW placentas (from 82 to 164 genes depending on the particular PLW litter size), we did not identify any genes encoding iron transporters or genes regulating placental iron transfer. To ensure that the expression of major genes of the iron transport machinery in the wild boar and domestic pig placenta is consistent, we next assessed their expression at the mRNA or protein levels, using real-time quantitative PCR or Western blot analysis, respectively. We found no differences in either TfR1 mRNA or L-Ft and Fpn protein levels. These results imply that both TfR1-mediated iron uptake and Fpn-mediated iron release are likely functioning at consistent levels across the conditions studied. Similarly, stable L-Ft protein levels indicate an equivalent iron status of all the analyzed placentas. Consequently, any alterations in iron homeostasis in this system may not be due to changes in the expression levels of these key iron transport proteins but rather might be regulated by other factors or mechanisms. Considering that DMT1 is likely involved in endosomal iron transport in placental cells [10,34], it is tempting to propose that the higher DMT1 protein level reported in the wild boar placenta may increase iron flow from the circulation of wild boars compared to that in PLW mothers. However, it should be remembered that within the endosome, DMT1 binds iron delivered by TfR1 [35], and as stated above, the ability of TfR1-mediated iron supply to the placenta was similar in females from all experimental groups. One of the most important findings of our study, attested first by RNA-seq and validated by real-time quantitative PCR, was the demonstration of the presence of highly expressed genes encoding multicopper ferroxidases such as ceruloplasmin (Cp), hephaestin (Heph), and zyklopen (Zp) [20,21] in wild boars compared to that in PLW placentas. These enzymes are Fe(II) oxidoreductases, whose structure is characterized by the presence of copper centers and a high-affinity ferrous iron binding site [20,21]. Importantly, all three ferroxidases have been reported to assist ferroportin in cellular iron efflux and its subsequent incorporation into transferrin for systemic distribution [17,36]. Multicopper ferroxidases are expressed in multiple tissues, but in individual tissues, each of them plays a specific role. Cp can be expressed as a secreted protein or as a membrane glycosylphosphatidylinositol-anchored protein (GPI-Cp) [37]. GPI-Cp is particularly required for iron efflux from cells in the central nervous system [36] and for efficient recycling of iron by macrophages, a process indispensable for systemic iron turnover [38]. Heph is a protein critical for intestinal iron absorption, highly expressed throughout the small intestine [29,39]. Zp has been identified as a placenta-specific ferroxidase, abundantly expressed in this tissue [28], although further research showed that it is not essential for the efficient transfer of iron to the fetus in mice [40]. To determine whether the increased expression of copper-related genes in the wild boar placenta is limited to multicopper ferroxidases, we examined the placental expression of other copper enzymes or copper-transporting proteins such as superoxide dismutase 1 (Sod1) [41] and P-type ATPases (Atp7a and Atp7b) [26]. Considering that copper is a potential factor regulating the expression of multicopper ferroxidases [27], we measured the content of this microelement in placentas but did not find any differences between the experimental groups. Interestingly, we observed an increased level of Atp7b transcript in wild boar placenta. This finding is related to the elevated Cp mRNA level in this placenta, as Atp7b ATPase is necessary for the metallation of apo-Cp, resulting in the formation of the redox-active enzyme holo-Cp [42]. In the next step of multicopper ferroxidase analysis in wild boar and PLW placentas, we used the immunofluorescence (IF) method and confocal microscopy to determine their localization and potential overlap in staining with ferroportin. We showed that both Cp and Zp are expressed in the maternal part of the placenta from all experimental groups, more precisely in the apical membrane of luminal epithelial cells of the endometrium, and they tightly co-localize with Fpn. Although Heph was also identified in several luminal epithelial cells of the endometrium, in contrast to Cp and Zp, it showed mainly basal localization with barely detectable (in wild boar females) or no co-localization (in PLW females) with Fpn. To our knowledge, the expression of multicopper ferroxidases in the placenta of *Sus* species has not been studied so far, and here we provide the first description of their expression and placental localization in wild boar and domestic pig. It should be noted that our understanding of pathways of iron transfer across the placenta is mostly based on human and rodent studies [4]. The mouse/human placenta is a hemochorial placenta, which means that maternal blood comes in direct contact with fetal trophoblasts [10]. Meanwhile, the pig placenta is classified as diffuse, mutually folded, non-invasive, and epitheliochorial, where there is no invasion of fetal tissue into the maternal endometrium [43]. From the maternal site, it is composed of the following layers: maternal endothelial vessels, endometrial connective tissue, endometrial luminal epithelium, trophoblast epithelium, chorionic connective tissue, and fetal endothelial vessels. The trophectoderm directly attaches to the endometria luminal epithelium, creating a bilayer at the maternal–fetal interface [43]. Nevertheless, despite anatomical differences in placenta structure between mammalian species, it seems that the functions of key proteins involved in iron transport through the placenta are similar. Although Cp and Heph have been identified in human [44] and mouse [45] placentas, there is evidence that these ferroxidases are not necessary for placental iron transport [20,46]. Our results showing extensive immunostaining of Cp in the apical membrane of luminal epithelial cells of the endometrium (especially in wild boar) and its tight co-localization with Fpn strongly suggest that Cp may facilitate iron egress from the maternal part of the placenta in the examined *Sus* species. In contrast, neither the intensity of Heph IF signals nor the distribution of Heph in the maternal part of the placenta indicate its active role in transplacental iron transport. Finally, IF analysis of Zp in wild boar and PLW placentas clearly shows thatm similarly to rodents [28], this ferroxidase may also be involved in placental iron metabolism in wild boar and domestic pig. Interestingly, besides finding Zp in the apical membrane of luminal epithelial cells of the endometrium (localization typical for its role in iron transport), we identified for the first time Zp protein expression in the nuclei of the luminal epithelial cells, exclusively in the basal part of the placental villi in both wild boar and PLW females. This particular localization of Zp may be associated with its role in cell proliferation as suggested previously [47].

In summary, our study emphasizes the lack of differences in the molecular potential to transfer iron across the placenta between domestic pig gilts having different-size litters. Furthermore, our results indicate that strong selection for a greater number of piglets in the litter does not result in simultaneous changes in placental iron metabolism. In gilts with large litters, the molecular iron transport machinery in the placenta is not adapted to satisfy the iron demand imposed by a greater number of fetuses. Furthermore, in PLW gilts, the ability for placental iron trafficking is reminiscent of that established in wild boars, the ancestors of domesticated pigs, which typically have less than half as many piglets per litter compared to domestic pigs. Importantly, our study provides evidence for the first time that all three multicopper ferroxidases are expressed in the maternal part of wild boar and PLW placentas, and two of them, namely Cp and Zp, seem to cooperate with Fpn in transporting iron to fetuses.

## 4. Materials and Methods

### 4.1. Animal Experimentation

The use of animals in the experiment and all procedures were approved by the Local Ethical Committee on Animal Testing at the Wrocław University of Environmental and Life Sciences (experiment on wild boar; permission number: 081/2020/P1) and the Second Local Ethical Committee on Animal Testing in Kraków (experiment on PLW pigs; permission no. 399/2020).

### 4.2. Polish Large White Pig 

The experiment with PLW pigs was conducted at the Pig Experimental Station in Żerniki Wielkie, belonging to the National Research Institute of Animal Production (Balice, Poland). The gilts were housed under standard conditions (70% humidity, temperature 22 °C) in gestation cages (2.2 × 0.65 × 1.8 m) with straw bedding. The gilts were placed in farrowing cages (2.4 × 3.4 m) on day 110 of gestation. Until parturition, the females were offered standard fodder for pregnant gilts containing 120 mg Fe/kg, as estimated by flame spectrometry. In this study, “large litter” refers to litters of 14 ± 2 piglets, contrasting with “small litters” of 4 ± 2 piglets. Although litters of 18–20 piglets are considered large in contemporary pig production [48,49], achieving such litter size through embryo transfer (ET) is challenging, especially in PLW pigs. Embryos were collected from fifteen 6-month-old PLW gilts (weighing 90–110 kg), superovulated with 1500 I.U. PMSG and 1000 I.U. hCG, then inseminated on the oestrus day with standard semen doses. The rmbryos were retrieved 72–74 h after hCG injection by flushing each oviduct with 20 mL of PBS with BSA at 38 °C. The embryos were then screened and evaluated morphologically. Recipient gilts (6-month-old PLW, ~90 kg) were synchronized with 750 I.U. PMSG and 500 I.U. hCG, followed by surgical ET on the second day after synchronized heat. Embryos (6–8 or 14–16 per recipient) were introduced into the fallopian tubes. Pregnancy success was confirmed by ultrasound, with large litters (10 ± 1 piglets) obtained from five gilts and small litters (4 ± 2 piglets) from seven gilts. Control large litters (14 ± 2 piglets) were obtained via routine artificial insemination (AI) from six 1-year-old sows. The control was used to assess the impact of the ET procedure on the outcomes of the studied parameters. This approach enabled a more accurate assessment of whether any differences in these parameters were due to the litter size rather than the technique used to produce the litters. The procedure has been described in detail [9].

### 4.3. Wild Boars

The experiment with wild boars was conducted at the Centre for Research into Forests and Game Breeding of Wrocław University of Environmental and Life Sciences (Złotówek, Poland). Clinically healthy wild boar females captured in the forest of Trzebnica and Oleśnica County, Lower Silesian Voivodeship, were kept in semi-wild conditions on a forest farm monitored by 12 cameras that were used to track their behavior, particularly rutting, mating, and preparations for farrowing. The farm was divided into three enclosures of approximately 1300 m^2^ each. Two sows were placed in each enclosure, and a male boar was introduced for the mating period (November to January). The animals had *ad libidum* access to water and were fed maize grain, potatoes, and fresh forage daily. The ground allowed for burrowing and other natural wild boar behaviors such as mud baths and scratching against tree trunks. For the experiment, we used six 1–2-year-old sows weighing 60–80 kg and having 4 ± 2 piglets in the litters [9].

### 4.4. Collection of Tissue Samples

Placental samples were collected from the maternal–chorioallantoic interface at each horn, immediately after its expulsion, then washed with PBS and immediately frozen in liquid nitrogen and stored at −80 °C prior to to RT-qPCR and Western blot analysis. Placenta samples for immunofluorescence analysis were fixed in 4% paraformaldehyde in PBS.

### 4.5. Next-Generation Sequencing

Total cellular RNA was extracted from tissues (20 mg) using an SV Total Isolation System (Promega, Madison, WI, USA) following the manufacturer’s instructions. RNA quality was controlled by a fluorescence-based quantification method and fragment-length analysis. After initial quality control, the needed samples were DNase-treated to remove residual DNA detected in them. Afterwards, all samples passed quality control and were further processed. The library preparation was carried out using a SMART-Seq Stranded kit from Takara Bio, (Kusatsu, Shiga, Japan). The total quantity used was 10.00 ng. RNA sequencing of 5 individuals per group was performed by CeGaT (Tubingen, Germany) using an Illumina NovaSeq 6000, 2 × 101 bp*. Q30 value of sequencing (RNA): ≥87.83%. Demultiplexing of the sequencing reads was performed using Illumina bcl2fastq (2.20). Adapters were trimmed with Skewer (version 0.2.2) [50]. Quality trimming of the reads was not performed. For samples prepared with the Takara kit (Kusatsu, Shiga, Japan) (SMART-Seq Stranded), the first three nucleotides of the second sequencing read (Read 2) were derived from the Pico v2 SMART adapter. Those three nucleotides were trimmed with Skewer (version 0.2.2) [50]. Trimmed raw reads were aligned to Sscrofa11.1 using STAR (version 2.7.3) [51]. Differential expression analysis between groups was performed with DESeq2 (version 1.24.0) [52] in R (version 4.0.4) (R Core Team 2015). Differential expression analysis between groups was performed with DESeq2 (version 1.24.0) [52] in R (version 4.0.4) (R Core Team 2015). DESeq2 uses a negative binomial generalized linear model to test for differential expression based on gene counts. Using normalized counts, DESeq2 calculates the log2 fold change, where the *p*-value reports the statistical significance of this result (Wald test). To identify genes with a significant difference in expression, a threshold of log2FoldChange ≥ 0.5 was applied. This means that genes were considered significantly altered if their expression ratio between groups differed by at least 50% (or its inverse) on a logarithmic scale. Since we compared thousands of genes, we had to correct for multiple testing. For this, we used the Benjamini–Hochberg correction [53] implemented in DESeq2 to adjust the *p*-value (padj). Genes with padj < 0.05 were considered statistically significant.

### 4.6. Real-Time Quantitative PCR

Total cellular RNA was extracted from tissues (20 mg) using an SV Total Isolation System (Promega, USA) following the manufacturer’s instructions. The concentration and purity of RNA were measured using a NanoDrop spectrophotometer (Thermo Scientific, Waltham, MA, USA). Two micrograms of total DNAse-treated RNA were reverse-transcribed using a Transcriptor First Strand cDNA Synthesis Kit (Roche, Basel, Switzerland). Real-time quantitative PCR analysis was performed in a LightCycler 96 (Roche Diagnostics, Mannheim, Germany) using gene-specific primer pairs (Table S1). The amplified products were detected using SYBR Green I (Roche Diagnostics, Switzerland). LightCycler 96 Software (Version 1.1.0.1320, Roche Diagnostics, Mannheim, Germany) was used for data analysis. Transcript levels were normalized relative to the ribosomal protein L4 (Rpl4). The analysis of RT-qPCR results was based on −ΔCt (cycle threshold) (Ct of reference gene—Ct of test gene). The control reference gene was selected using NormFinder software (https://www.moma.dk/software/normfinder (accessed on 9 July 2024)). NormFinder identifies the most stable reference genes by assessing expression variance between and within sample groups to ensure precise normalization in gene expression studies.

### 4.7. Protein Extract Preparation and Western Blotting 

For the analysis of proteins, placental tissues were homogenized in a sucrose–histidine buffer (0.25 mol/L sucrose, 0.03 mol/L histidine, pH 7.2) supplemented with protease inhibitors (Sigma-Aldrich, Saint Louis, Missouri, USA). The homogenates were centrifuged at 6000× *g* for 15 min at 4 °C. The supernatant was transferred to new centrifuge tubes and subjected to ultracentrifugation at 36,000 rpm using an Optima XPN-100 ultracentrifuge (Beckman Coulter, Brea, California, USA) for 45 min at 4 °C to separate membrane and cytoplasmic protein fractions. The cytoplasmic protein fraction, obtained from the supernatant, was frozen, while the membrane protein fraction, found in the sediment, was resuspended in the sucrose–histidine buffer. To obtain the total protein fraction, homogenates were centrifuged in a sucrose–histidine buffer at 12,000× *g* for 20 min at 4 °C, and the pellet was discarded. The resulting suspension was stored at −80 °C. Protein concentrations were determined using the Bradford assay (Bio-Rad, Hercules, California USA), and protein extracts were subjected to SDS-PAGE electrophoresis. Transfer efficiency was verified by Ponceau-S staining. A PVDF membrane was blocking with 5% skimmed milk. The details of primary and secondary antibodies used are provided in Table S2. After incubation with the primary antibody overnight at 4 °C, the blots were washed and incubated with secondary antibodies at room temperature for 1 h, followed by visualization using the SuperSignal™ Western Blot Enhancer (Thermo Scientific, USA). Band intensities were quantified by densitometric analysis using Quantity One software, version 4.6.6 (Biorad, Hercules, CA, USA). For our Western blot analysis, β-actin was employed as a loading control to ensure the accuracy and reliability of protein quantification.

### 4.8. Measurement of Copper Content in Placenta

Copper concentration was determined in the placenta by atomic absorption spectroscopy. Samples were digested in 2 mL of boiling Suprapur-grade nitric acid (Merck, Darmstadt, Germany). After cooling to RT, each sample was suspended in 10 mL of deionized water. Reference material samples were prepared in a similar manner. The copper concentration was measured using the graphite furnace atomic absorption spectrometry (AAS) technique (AAnalyst 800, PerkinElmer, Waltham, MA, USA).

### 4.9. Immunofluorescence (IF) Analysis and Confocal Microscopy of Placental Sections

Placentas were immediately dissected and fixed in 4% paraformaldehyde (MilliporeSigma, Burlington, MA, USA) in phosphate-buffered saline (PBS) at 4 °C for 24 h. Following two 30-min washes in PBS, the placentas were successively soaked in 12.5% and 25% sucrose (Bioshop, Burlington, ON, Canada) at 4 °C for 2 h and 7 days, respectively. The placental samples were then embedded in a Cryomatrix medium (Thermo Scientific, USA), frozen in liquid nitrogen, and sectioned in 20 μm slices using a cryomicrotome (Leica, Wetzlar, Germany). The sections were washed in PBS for 10 min and permeabilized by bathing in PBS/0.1% Triton X-100 (MilliporeSigma, Burlington, MA, USA) for 20 min. Non-specific antibody binding was blocked by incubating the tissue sections in PBS/3% BSA (Bioshop, Canada) at room temperature (RT) for 1.5 h. For protein detection, sections were incubated overnight at RT with primary antibodies diluted in PBS/3% BSA. The primary and fluoro-chrome-conjugated secondary antibodies used in IF analysis are described in Table S2. Next, the sections were washed for 5 × 6 min with PBS/0.1% Triton X-100 and incubated for 1.5 h with secondary antibody diluted in PBS/3% BSA at RT. Finally, the sections were washed for 10 min in PBS and mounted in a Vectashield medium with DAPI (Vector Labs, Newark, CA, USA). 

The presence of ferroxidases and Fpn in the luminal cells of the endometrium was determined by double immunofluorescence localization of the investigated proteins. In order to distinguish different proteins in this experiment, the secondary antibodies were conjugated with different fluorochromes: Cy3 for Cp and Zp, Alexa488 for Fpn, Alexa488 for Heph, and Cy3 for Fpn. For the immunolocalization of Fpn and ferroxidases, the placenta sections were first incubated with anti-Fpn and then with anti-ferroxidases primary antibodies. Then, the sections were incubated with the mixture of secondary antibodies. The sections were then washed for 5 × 6 min with PBS/0.1% Triton X-100 and for 10 min in PBS and mounted in a Vectashield medium with DAPI (Vector Labs, Newark, CA, USA). IF was analyzed with a Zeiss LSM 710 confocal microscope (CarlZeiss, Oberkochen, Germany) using a ×40 objective and Zeiss ZEN software (version 3.0).

### 4.10. Statistical Analyses

The results are presented as the means ± SD (standard deviation). Kolmogorov–Smirnov tests were performed to evaluate normality. One-way analysis of variance (ANOVA) was used for statistical evaluation of the data, and post hoc Tukey’s test was applied (*p* < 0.05).

## 5. Conclusions

The results of our study challenge the idea that domestic pig females can adapt to meet the increased iron demand imposed by a high number of piglets in a litter by enhancing transplacental iron transfer. Essentially, domestic pig females retain the molecular potential for iron transfer across the placenta, a trait inherited from their ancestor, the wild boar. Although the analysis of major iron transporters did not show significant differences, the higher expression of copper-dependent ferroxidases in wild boar placentas suggests that their presence may influence the efficiency of iron export to the fetal circulation. The extent to which this phenomenon contributes to meeting the iron requirements of larger litters requires further research.

## Figures and Tables

**Table 1:** Differentially expressed genes (DEGs) in the placentas of wild boars and PLW pigs.

Comparison between Experimental Groups	Number of Significantly Changed Genes		
	Total	Upregulated	Downregulated
Small litter (ET) vs. wild boar	164	138	26
Large litter (ET) vs. wild boar	402	94	308
Large litter (AI) vs. wild boar	82	46	36
Large litter (ET) vs. small litter (ET)	0	0	0
Large litter (AI) vs. small litter (ET)	1	0	1
Large litter (AI) vs. large litter (ET)	2	1	1

ET—embryo transfer, AI—artificial insemination, padj < 0.05 adjusted *p*-value.

**Table 2:** Changes in the expression of ferroxidase genes in the placenta of wild boar and PLW females.

Gene Names									
Comparsion between Experimental Groups	Hephaestin (*Heph*)			Ceruloplasmin (*Cp*)			Hephaestin like 1 (*Hephl1*)		
	Log2-Fold Change	*p*	padj	Log2-Fold Change	*p*	padj	Log2-Fold Change	*p*	padj
Large litter (AI) vs. wild boar	−0.52	0.17	0.72	−0.98	0.22	0.78	−1.48	0.23	0.78
Large litter (ET) vs. wild boar	−0.73	0.05	0.33	−0.32	0.69	0.90	−3.40	0.01	0.11
Small litter (ET) vs. wild boar	−0.34	0.40	0.74	−0.99	0.25	0.64	−3.80	0.00	0.13
Large litter (ET) vs. small liter (ET)	−0.39	0.33	1.00	0.67	0.44	1.00	0.40	0.76	1.00
Large litter (AI) vs. small litter (ET)	−0.18	0.65	1.00	0.00	1.00	1.00	2.32	0.08	0.89
Large litter (AI) vs. large litter (ET)	0.21	0.58	1.00	−0.66	0.41	1.00	1.91	0.12	1.00

ET—embryo transfer, AI—artificial insemination, *p*—*p*-value, padj—adjusted *p*-value.

## Data Availability

The authors declare that the data supporting the findings of this study are available within the paper.

## Supplementary Materials

The following supporting information can be downloaded at: https://www.mdpi.com/article/10.3390/ijms25179638/s1.
