# Supplementary material for: Comparison of Molecular Potential for Iron Transfer across the Placenta in Domestic Pigs with Varied Litter Sizes and Wild Boars
Source: Int J Mol Sci. 2024 Sep 5;25(17):9638. doi: 10.3390/ijms25179638 (PMC11395084; doi:10.3390/ijms25179638)
Supplement: Supplementary file 1 [file ijms-25-09638-s001.zip › ijms-3158644-supplementary.pdf]

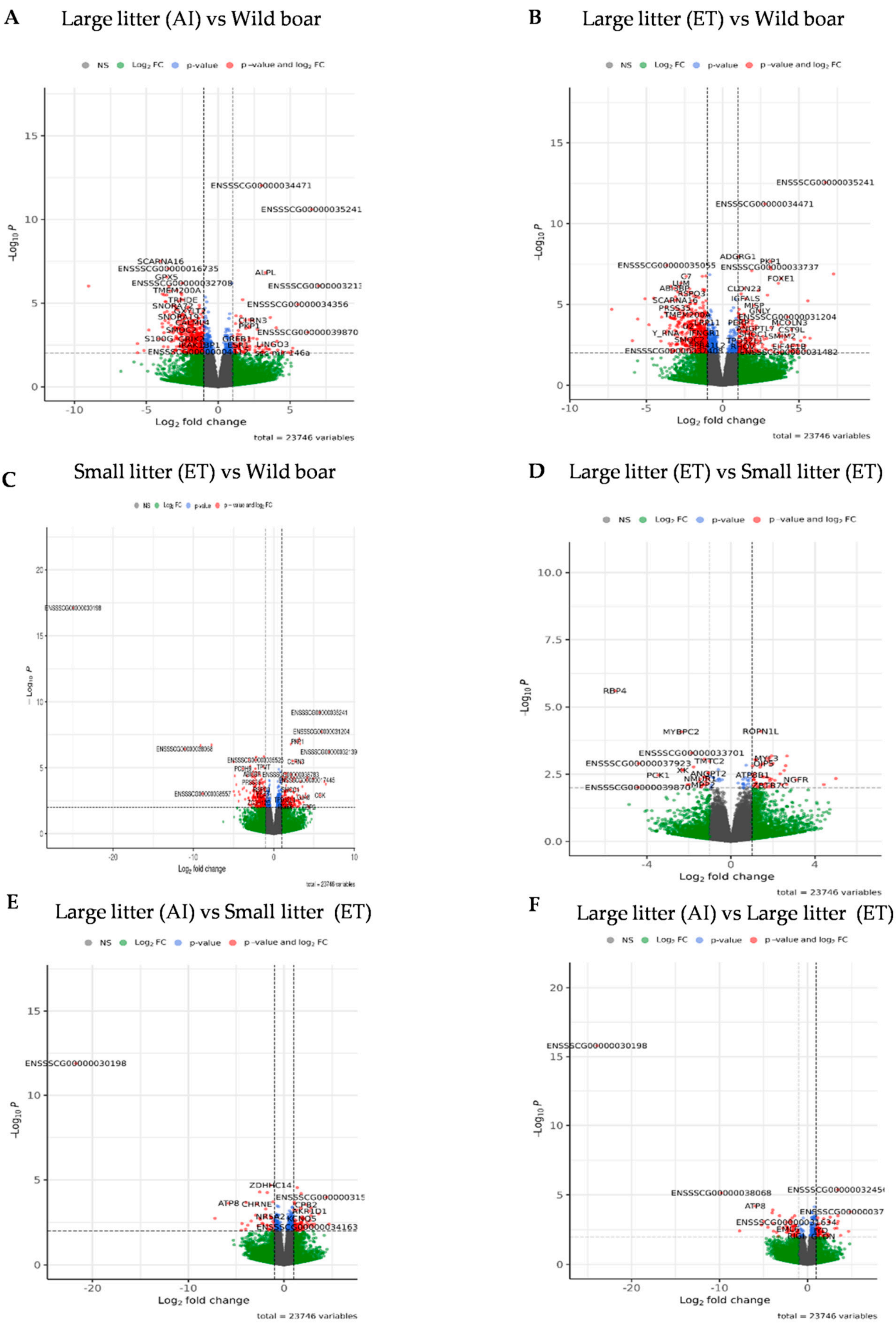

Figure S1. Volcano plots showing differentially expressed genes' comparison in the placentas with (**A**) the large litter obtained by artificial insemination (AI) vs wild boar, (**B**) large litter obtained by embryo transfer (ET) vs wild boar, (**C**) small litter (ET) vs wild boar, (**D**) large litter (ET) vs small litter (ET), (**E**) large litter (AI) vs small liter (ET) and (**F**) large litter (AI) vs large litter (ET). The most variable genes are identified by red dots.

Table S1. List of primers for real-time quantitative PCR.

Gene	Forward primer (5′→3′)	Reverse primer (5′→3′)
Fpn	TCGCCTAGTGTCATGACCAG	CAGAAACACAGACACCGCAA
Tfr1	GCCGAAGTGGCTGGTCATC	CAGACTCAGACCCATCTCCCT
Dmt1	GCAGGTGGTTGACGTCTGTA	CACGCCCCCTTTGTAGATGT
Lft	TGTTTGGACGGAACAGACCC	CTCATGGCTGGTCGGCAATA
Rpl4	CTCAGCGAATGAGAGCTGGT	GTGTTTCGGCGCATTGTCTT
Cp	AGGTCCACAACTTCATGCCG	GTGCGTGTTTCACCTGGTACT
HephL	CTGTGCTTACCCACGTTGTC	GAAGGAAGGGACAAGGTCA
Heph	TCGCATCTTTCCAGCCACCT	GTGCTTGCATGCCACCTTTC
Atp7b	TGGGATCGGCTGCAAAGTCA	GGCCGCATCTGTTTCTGAGG
Sod1	AAGGCCGTGTGTGTGCTGAA	TGCACTGGTACAGCCTTGTGT

Table S2. List of antibodies.

[illegible]	protein	[illegible]	dilution	[illegible]	dilution
Western blotting	Fpn	FPN1 Polyclonal antibody, #PA5-22993 (Invitrogen)	1:1000	Goat anti-rabbit polyclonal, #A6154 (Sigma-Aldrich)	1:10000
	β-Actin	Mouse monoclonal, #MA5-11869, (Thermo Fisher)	1:1000	Goat anti-mouse polyclonal, #A5278 (Sigma-Aldrich)	1:10000
	L-Ft	Rabbit polyclonal, #ab69090, (Abcam)	1:1000	Goat anti-rabbit polyclonal, #A6154 (Sigma-Aldrich	1:10000
	Cp	Goat-anti human, #C0911, (Sigma- Aldrich)	1:1000	Donkey anti-goat, polyclonal, #sc-2020 (Santa Cruz Biotechnology)	1:10000
	DMT1	Rabbit polyclonal, #NRAMP22-A, Alpha Diagnostic,	1:1000	Goat anti-rabbit polyclonal, #A6154 (Sigma-Aldrich)	1:1000
Immunofluorescence (IF) staning	Heph	Mouse monoclonal, Santa Cruz Biotechnology), sc-365365	1:100	Goat anti-mouse conjugated with Alexa488® fluorochrome (Jackson Immunoresearch)	1:500
	Cp	Rabbit polyclonal anti-Cp antibody (Dako) Q0121	1 : 100	Goat anti-rabbit conjugated with Cy3® fluorochrome (Jackson Immunoresearch)	1:500
	Zp	Rabbit polyclonal, US Biological, No. 036510	1:100	Goat anti-rabbit conjugated with Cy3® fluorochrome (Jackson Immunoresearch)	1:500
	Fpn	Goat polyclonal, Invitrogen, #PA5-143047	1:150	Donkey anti-goat conjugated with Alexa Fluor Plus 488 fluorochrome (Invitrogen)	1:500
	Fpn	Rabbit polyclonal, Alpha Diagnostic , #MTP11-A	1: 150	Goat anti-rabbit conjugated with Cy3® fluorochrome (Jackson Immunoresearch)	1:500
